# Use of Arsenic for the Relief of Asthma

**Published:** 1874-03

**Authors:** Comegys Paul


Use of Arsenic for the Relief of Asthma.—*By Comegys Paul, M.D.*—A thorough sympathy for this class of unfortunates has induced me to make experiment of nearly all the drugs recommended by good authorities. The beneficial effects of many of them are confined to a limited number of sufferers. Some certainly afford palliation, imperfect though it may be, while few can be said to exercise any influence for good after the exacerbation has passed. As a prophylactic no particular remedy can be confidently recommended, but, from my own experience, I am able to advocate the use of *arsenic* as affording more satisfaction than any one drug that I have made trial of; indeed, I am inclined to think that it is the remedy *par excellence*, not only for the mitigation of spasmodic asthma, but also as a reliable propylactic.

From the absence of anatomical lesions after death, and from the suddenness of the attack and its quick subsidence, we know that it is not of an inflammatory nature, and are led with much certainty to regard it as of purely nervous origin. Therefore, when it is not connected with bronchitis, should we not, with much reason, rely entirely upon drugs which are known to affect the nervous centers. If we except cases caused by indigestion, I am confident that arsenic, properly given, will afford much relief. The peculiar excellence of belladonna as a sedative in asthma, I believe to be similar in its action to that of arsenic, by its diminution of reflex irritability.

To be efficacious it must be pushed until it gives decided evidence of its presence in the patient's system. I have seen it relieve sufferers after all the other recognized drugs have failed. Cases that did not yield in the beginning, have done so by a continuation of its use, assisted by medicine to counteract indigestion, bronchitis, etc. I have arrested an excessive dyspnœa, resulting from an aggravated attack of asthma, almost instantaneously by the use of Fowler's solution hypodermically. By instantaneously I mean with the same rapidity that a proper dose of morphia, similarly given, will relieve pain.

As a prophylactic, the liq. potas. arsenitis should be given in five-drop doses three times a day, to be increased or diminished as symptoms may indicate. When the patient is suffering at the same time with anæmia, or any wasting disease, it may be combined advantageously with the citrate of iron and quinia. Ordinary cases of asthma will generally be influenced by this treatment in the course of three or four days. Bad cases of years' standing have commonly yielded their frequency and violence after three days, and very seldom are any entirely unbenefited after a week's exhibition. When given before the commencement of an autumnal catarrh, it will often prevent its onset, and the exceeding discomfort attendant upon this form of asthma can be controlled so long as the remedy is persisted in.

